# Successful Management of Metastatic Eccrine Porocarcinoma

**DOI:** 10.1155/2013/282536

**Published:** 2013-10-26

**Authors:** Imane Aaribi, Amina Mohtaram, Meryam Ben Ameur El Youbi, Jinane Kharmoum, Mustapha El Kabous, Hind Mrabti, Basma El Khannoussi, Hassan Errihani

**Affiliations:** ^1^Department of Medical Oncology, National Institute of Oncology, Rabat, Morocco; ^2^Department of Pathology, National Institute of Oncology, Rabat, Morocco

## Abstract

Eccrine porocarcinoma (EPC) is a rare tumor. It develops from the intraepidermal ductal portion of the eccrine sweat gland. Metastatic disease is rare. We report a new case of metastatic eccrine porocarcinoma with a successful management and a good response to docetaxel. A 54-year-old man was admitted with a mass in the breast. Biopsy specimen found carcinomatous tumor proliferation with large anastomosing ducts. Cellular atypia were noted, with eosinophilic cytoplasm and round to oval nuclei. The tumor showed positive immunoreactivity for ACE and negative to anti-PS-100. Resection was performed. One year later, he presented with local and metastatic recurrences. The patient had received 3 cycles of cisplatin and 5-fluorouracil; he progressed with increase in mass size and number of lung lesions. He has been undergoing three cycles of docetaxel with complete response in the lung and regression of the breast mass. The mass was excised. Porocarcinoma is a very rare entity and poorly understood. In the metastatic phase, it has modest or no sensitivity to anticancer treatment. Docetaxel should be considered in the metastatic eccrine porocarcinoma.

## 1. Introduction

Eccrine porocarcinoma (EPC) is a rare tumor [1]. It develops from the intraepidermal ductal portion of the eccrine sweat gland [2]. This tumor was initially described by Pinkus and Mehregan as epidermotropic eccrine carcinoma [3, 4]. Metastasis occurred infrequently. When it occurs, it's habitually visceral [5]. Few therapeutics are effective in their disseminated form [6]. We report a new case of metastatic eccrine porocarcinoma with a successful management and good response to docetaxel. 

## 2. Case Report

**Figure 1:**
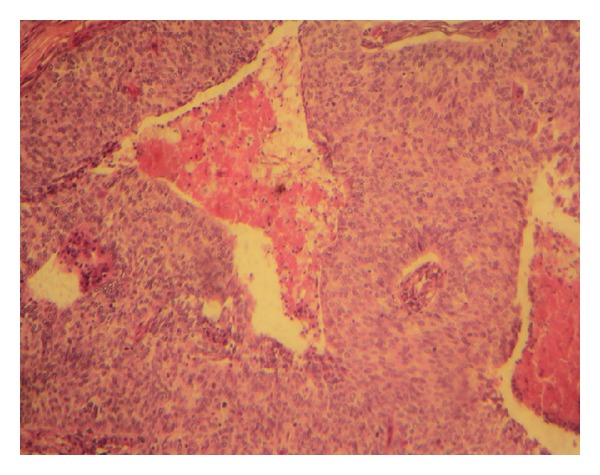
Large anastomosing ducts containing cell nests beneath ulcerated surface (hematoxylin-eosin staining, original magnification ×10).

**Figure 2:**
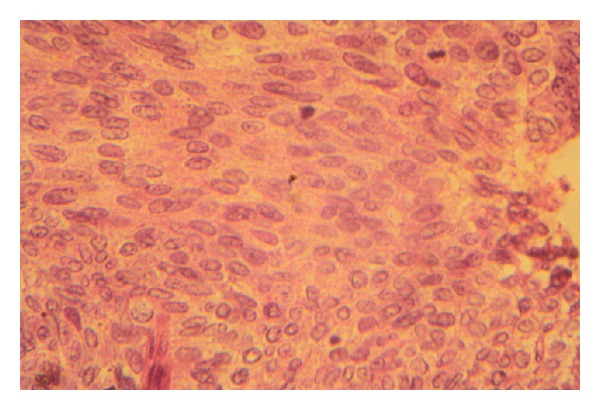
Microscopic findings showing cytonuclear atypia (hematoxylin-eosin staining, original magnification ×40).

**Figure 3:**
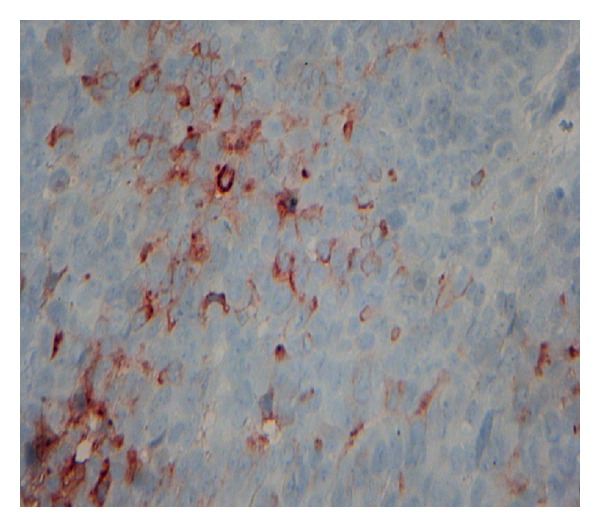
Staining of tumor cells with antibody to carcinoembryonic antigen confirming their eccrine differentiation (original magnification ×40).

A 54-year-old patient was admitted with a mass in the breast, the biopsy specimen found carcinomatous tumor proliferation with ulcerated surface, large anastomosing ducts that contained clear cell nests (Figure 1). Cellular atypia were noted, with eosinophilic cytoplasm and round to oval nuclei (Figure 2). The tumor showed positive immunoreactivity for AE1/AE3 and ACE (Figure 3) and negative to CK5/6 and PS 100. Based on these findings, the tumor was identified as adnexal tumour, eccrine porocarcinoma. Resection was performed. One year later he presented with local and metastatic recurrence. Computed tomography scan discovered metastatic lesions in the lung. The patient had received 3 cycles of cisplatin and 5-fluorouracile, he progressed with increase in size and number of lung lesions. He has been undergoing second line chemotherapy docetaxel. He received three cycles with complete response in the lung and regression at the breast mass. The mass was excised.

## 3. Discussion

Eccrine porocarcinoma is a rare tumor [1]. It accounts for 0.005–0.01% of all cutaneous tumors [3]. It occurs in the elderly, usually after 60 years [1, 2, 6]. It can be primary or occur on an evolving benign eccrine poroma [1]. The predilection site is the lower extremities (55%), followed by the head and scalp (20%), upper limbs (12%), and trunk and abdomen (10%) [7]. It has no clinical features. It can appear as solitary plaque or nodular lesion, with ulcerated or hemorrhagic surface [1].

The differential diagnosis with other malignant tumors of the skin is very complex, especially with seborrheic keratosis, Bowen tumor, multifocal basal cell carcinoma, lymphoma, achromic melanoma, pyogenic granuloma, wart, and nevus.

The classic histological description of eccrine porocarcinoma is an acanthotic epithelial proliferation that contained clear cell nests with radial extension of polygonal nuclei, eosinophilic cytoplasm, and rudimentary ductal structures with many intraepidermal atypia [6]. The diagnosis is based on morphology rather than immunohistochemistry, there may be an expression of CEA and EMA; PS-100 is negative. These markers confer variable results and do not confirm the diagnosis [8].

Metastasis occurs in about 20% of cases with a very poor outlook and high mortality. They occurred preferentially in lymph nodes, lung, retroperitoneum, and liver [8].

 The main treatment for localized form is surgical excision with histologically clear margins, [9, 10]; it's the treatment of choice. Electrocoagulation and radiotherapy can be performed but with high risk of local recurrence. In the metastatic eccrine porocarcinoma, treatment with tamoxifen or retinoid may have led to remissions of several months [6]. The chemotherapy and radiotherapy are ineffective with uncertain benefit [7].

Single agent docetaxel have been used in metastatic eccrine porocarcinoma with symptomatic and radiological response lasting several months. It was well tolerated [11].

Our patient had complete response in the lung and regression of the breast mass after three cycles of docetaxel. Docetaxel should be considered in platinum resistant or refractory patients with metastatic eccrine porocarcinoma.

## 4. Conclusion

Porocarcinoma is a very rare entity and poorly understood. The clinical diagnosis is very evocative. The standard pathological diagnosis establishes the diagnosis easily. Immunohistochemistry is useful in difficult cases. Treated early, eccrine porocarcinoma is curable by wide excision. In the metastatic phase, it has little or no sensitivity to anticancer treatment. Docetaxel should be considered in platinum resistant or refractory patients with metastatic eccrine porocarcinoma.
